# “Simple mechanical devices did not improve pelvis positioning in AP pelvis radiographs for reliable assessment of the acetabular orientation”

**DOI:** 10.1186/s40634-019-0191-7

**Published:** 2019-05-23

**Authors:** Matthias Brockmeyer, Sebastian Lott, Jonas Stroeder, Peter Fries, Stefan Wagenpfeil, Thomas Tschernig, Dieter Kohn

**Affiliations:** 1 Department of Orthopaedics and Orthopaedic Surgery, Saarland University Medical Center, Kirrberger Straße, Bdg. 37, 66421 Homburg/Saar, Germany; 2 Department of Diagnostic and Interventional Radiology, Saarland University Medical Center, Kirrberger Straße, Bdg. 50.1, 66421 Homburg/Saar, Germany; 3 Institute of Medical Biometry, Epidemiology and Medical Informatics, Saarland University Medical Center, Kirrberger Straße, Bdg. 86, 66421 Homburg/Saar, Germany; 4 Institute of Anatomy and Cell Biology, Saarland University Medical Center, Kirrberger Straße, Bdg. 61, 66421 Homburg/Saar, Germany

**Keywords:** Femoroacetabular impingement (FAI), AP pelvis radiograph, AP pelvis x-ray, Acetabular orientation, Anterior pelvic plane (APP), Pelvic tilt

## Abstract

**Background:**

The aim of this study was to develop two simple positioning devices for anteroposterior pelvis radiographs and to evaluate their effect on accuracy of the radiographs for assessment of the acetabular orientation compared with non-instrumented positioning.

**Methods:**

The superior anterior iliac spines and the pubic symphysis were used as anatomical landmarks to obtain a horizontal orientation of the pelvis according to the anterior pelvic plane. Anteroposterior pelvis radiographs were taken of 11 human cadaveric pelvic bones with each of the positioning devices and without any device. Defined measurements were carried out to objectify the tilt and rotation of the pelvis and to assess the cross-over sign as well as the presence of the ischial spine sign. Computed tomography scans were performed as a standard of reference. Bland-Altman-Plots were used to compare the continuous measurement values and Cohen’s Kappa was applied for the categorical data. Intra- and inter-observer reliability was determined by the intraclass correlation coefficient and Cohen’s Kappa.

**Results:**

The mean values of the measurements showed a high variability. A low correlation of the measurement values was found between the radiographs of the different positioning methods and the computed tomography scans. The intra- and inter-observer reliability was good (Cohen’s Kappa 0.78 and 0.69; intraclass correlation coefficient 0.99 and 0.98).

**Conclusion:**

The use of positioning devices did not lead to more accurate anteroposterior pelvis radiographs compared to non-instrumented positioning. Simple positioning devices do not provide standardized anteroposterior pelvis radiographs for reliable assessment of the acetabular orientation.

## Background

In the assessment of the spatial acetabular orientation, it is crucial that the bony rim of the hip socket is correctly identified. It is clinically relevant for the evaluation of the correct reorientation of the acetabulum after periacetabular osteotomy and for the exact analysis of the acetabular orientation in cases of femoroacetabular impingement (FAI) type pincer (Reynolds et al. 1999). In the latter cases, a positive cross-over sign (COS) (Jamali et al. 2007) or posterior wall sign (PWS) (Tannast et al. 2007) and the presence of the ischial spine sign (ISS) (Kakaty et al. 2010) are radiographic indicators for a retroversion of the acetabulum (Reynolds et al. 1999). Conventional radiographic diagnostics has remained very important despite imaging by computed tomography (CT) and magnetic resonance (MR). It is cost effective, fast, easy to use and readily available. For precise measurements on radiographs an accurate positioning is a precondition. Anteroposterior (AP) pelvis radiographs are influenced significantly by incorrect pelvic positioning regarding pelvic tilt and rotation. This can lead to false-positive or false-negative radiographic findings and in some cases to wrong diagnoses (Anderson et al. 2012; Clohisy et al. 2009; Dandachli et al. 2009; Jamali et al. 2007; Siebenrock et al. 2003; Tannast et al. 2005). Wassilew et al. (Wassilew et al. 2012) reported that the COS and the PWS determined from AP pelvis radiographs are influenced by pelvic tilt and they concluded that AP radiographs do not provide reliable radiographic findings for the assessment of acetabular retroversion. Simple positioning devices that help to adjust the pelvic orientation according to the APP concept (Lewinnek et al. 1978; McKibbin 1970) for AP pelvis radiographs might improve the accuracy and lead to a higher degree of standardization. The aim of this study was to develop simple positioning devices for AP pelvis radiographs and to evaluate their effect on standardization and accuracy of the radiographs for reliable assessment of the acetabular orientation compared with the non-instrumented, standard method of pelvic positioning. Corresponding CT scans were performed as standard of reference. We hypothesized that the use of positioning devices for AP pelvis radiographs leads to more accurate and standardized radiographs for reliable assessment of the acetabular orientation compared to the non-instrumented positioning technique.

## Methods

### Specimens

Eleven human cadaveric bony pelves of embalmed body donors (6 male, 5 female) were used in this study (diagnostic study, level of evidence: II). The soft tissue structures were completely resected and the femora were removed. The mean age of the body donors at the time of death was 79.8 [60–92] years. Bodies with previous injury or surgical procedures of the hip joints and the pelvis as well as specimens with signs of severe osteoarthritis were excluded. The specimens were obtained from the body donation program of Saarland University Medical Center, Institute of Anatomy and Cell Biology.

### Positioning techniques

**Fig. 1:**
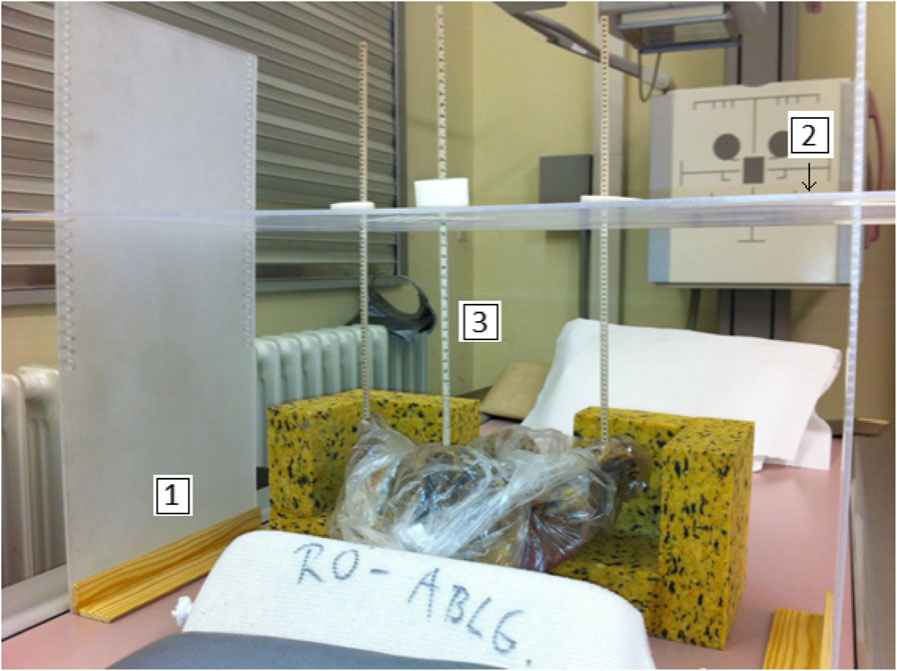
Positioning device I: vertical acrylic glass panel attached to wooden base (1), height adjustable horizontal acrylic glass panel (2) with guide slots for the styli (3). The styli were placed in the same height on the superior anterior iliac spines and the pubic symphysis

**Fig. 2:**
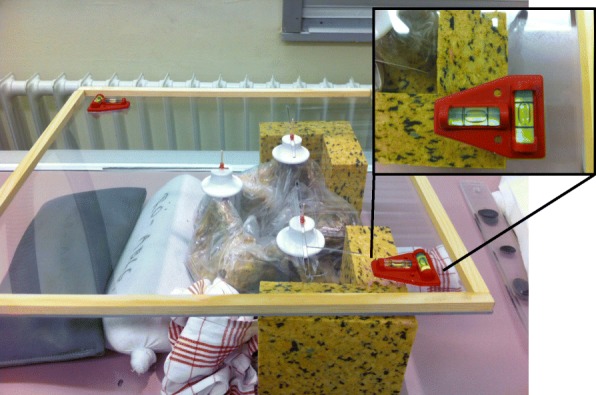
Positioning device II: a wood-framed acrylic glass plate with three different guide slots that allowed for individual positioning of the pegs; the pegs were placed on the superior anterior iliac spines and the pubic symphysis; additional spirit levels (insert) were installed for the horizontal alignment

Two simple positioning devices for standardized AP pelvis radiographs were developed and tested. Radiolucent and rigid materials were used for the construction of the devices. The anterior facet of the superior anterior iliac spines and the anterior surface of the pubic symphysis were used as anatomical landmarks to obtain a horizontal orientation of the pelvis according to the APP. Positioning I was performed with the help of a device which was composed of two vertical acrylic glass panels with wooden bases and one height adjustable horizontal acrylic glass panel. The latter panel had different guide slots for three wooden styli. The height of the styli was marked visibly in millimeters. The styli were placed on the superior anterior iliac spines and the pubic symphysis and fixed on the same height to ensure an exact horizontal orientation according to the APP. Additional foam pads and sandbags were used for a more stable position of the bony pelvis on the x-ray table (Fig. 1). Positioning II was done by an instrument which was constructed of a wood-framed acrylic glass plate which had three different guide slots for variably adjustable pegs. The pegs were also placed on the superior anterior iliac spines and the pubic symphysis to ensure a correct orientation according to the APP. Additionally, this instrument was equipped with two spirit levels to control horizontal alignment. According to positioning technique I, foam pads and sandbags were utilized to obtain a stable pelvis position on the x-ray table (Fig. 2). Positioning III was a non-instrumented technique. The pelvic specimens were placed by visual judgement to achieve a horizontal orientation of the pelvis according to the APP. Foam pads and sandbags were also used to stabilize the bony pelvis on the x-ray Table.

### AP pelvis radiographs

**Fig. 3:**
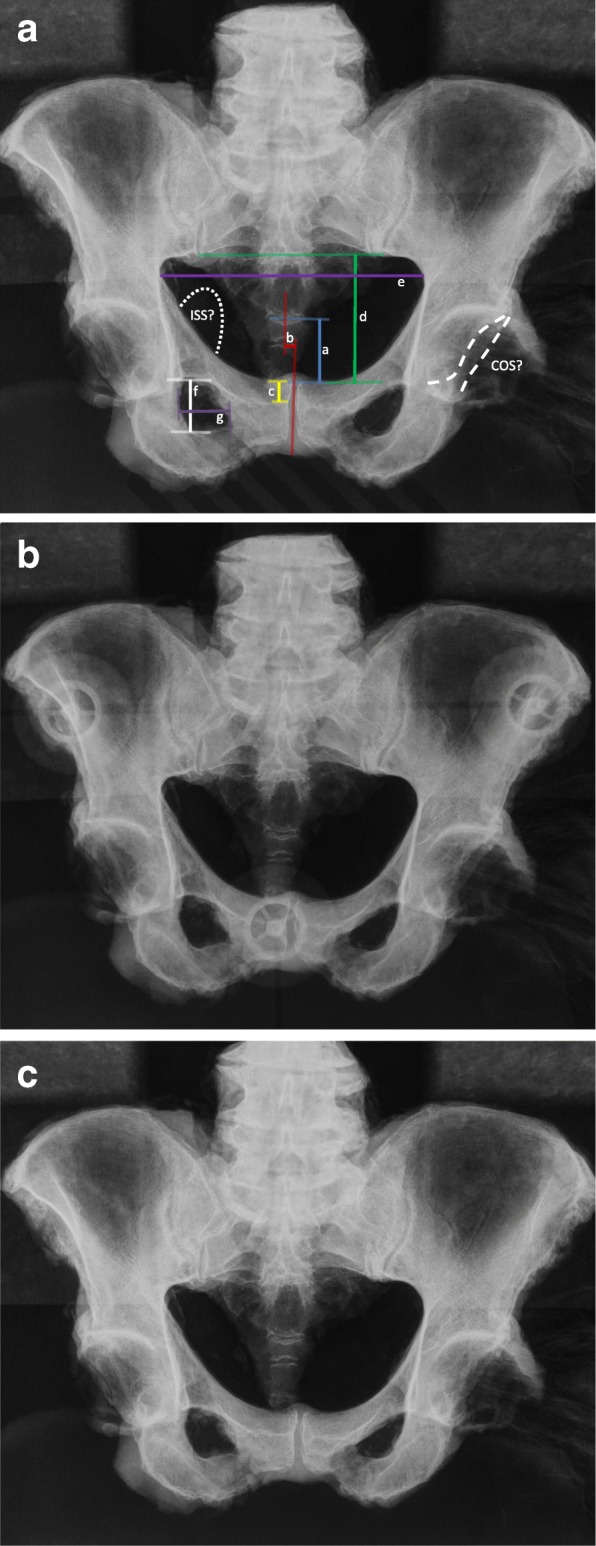
AP pelvis radiographs of the same cadaveric pelvic specimen with: **a** positioning I, **b** positioning II, **c** positioning III (ISS: ischial spine sign; COS: cross-over sign; a, bold blue line: vertical distance between the upper boarder of the symphysis and the sacrococcygeal joint; b, bold red line: horizontal distance between the midpoint of the symphysis and the midpoint of the sacrococcygeal joint; c, bold yellow line: vertical distance between the upper boarder of the symphysis and the tip of the coccyx; d, bold green line: vertical distance between the upper boarder of the symphysis and a line connecting the lower ends of the sacroiliac joints; e, bold purple line: horizontal diameter of the pelvic foramen; f, bold white line: vertical diameter of the obturator foramen; g, bold brown line: horizontal diameter of the obturator foramen)

AP pelvis radiographs were acquired using a clinical x-ray machine (Philips Optimus®, Best, Netherlands) from all cadaveric pelvic bones with each of both positioning devices (positioning I and II) and in the described non-instrumented positioning method (positioning III). A source-to-image distance of 110 cm was established for all radiographs. The central beam of the radiograph was adjusted orthogonal to the center of the symphysis. The radiographic magnification was defined as 110%. Defined measurements were carried out to objectify the tilt and rotation of the pelvis according to Tannast et al. (Tannast et al. 2006) and to assess the COS as well as the presence of an ISS as indicators for acetabular retroversion. The following measurement parameters were analyzed: (1) vertical distance between the symphysis and the sacrococcygeal joint, (2) horizontal distance between the symphysis and the midpoint of the sacrococcygeal joint, (3) vertical distance between the symphysis and the tip of the coccyx, (4) vertical distance between the symphysis and a line connecting the lower ends of the sacroiliac joints, (5) ratio between the vertical and horizontal diameter of the pelvic foramen and (6, 7) ratio between the vertical and horizontal diameter of the obturator foramen on both sides (Fig. 3).

### CT scans

**Fig. 4:**
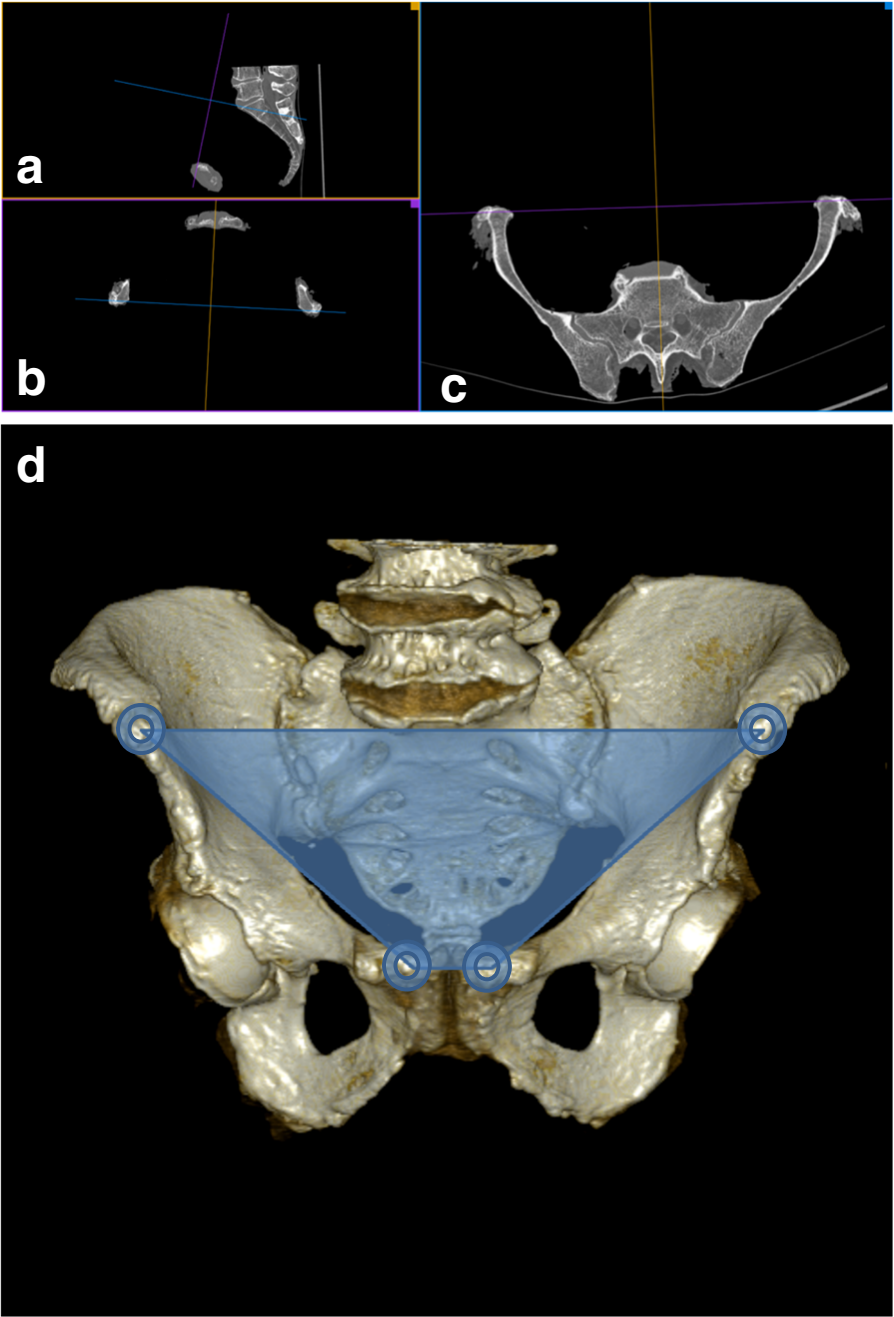
The pelvic orientation was exactly adjusted to the APP in all spatial planes. **a** Adjusted sagittal reconstruction. **b** Adjusted coronal reconstruction: this is the APP connecting the upper boarder of the pubic symphysis and the anterior facet of the superior anterior iliac spines. **c** Adjusted sagittal reconstruction. (purple line: adjusted coronal plane, APP; blue line: adjusted axial plane; yellow line: adjusted sagittal plane) **d** CT 3D reconstruction of a pelvic specimen adjusted to the APP (blue trapezoid)

CT scans were performed of all pelvic specimens as a standard of reference by a 16-slice spiral CT scanner (Philips Brilliance 16®, Best, Netherlands). A tube voltage of 120 kV with a tube current of 300 mAs was applied for each scan. The reconstruction of the images was performed in a bone window setting with a slice thickness of 0.8 mm and an increment of − 0.4 mm and in a soft-tissue window setting with a slice thickness of 3 mm and an increment of − 2 mm. The CT images were saved in a DICOM (Digital Imaging and Communication in Medicine) format and were transferred to a dedicated workstation running Osirix MD 7.5 (Pixmeo SARL, Geneva, Switzerland). For the analysis, the pelvic orientation was exactly adjusted to the APP (Fig. 4).

The acetabular version was determined in the adjusted axial plane according to the technique described by Dandachli et al. (Dandachli et al. 2011). It was defined as the angle formed by a reference line that was perpendicular to a line connecting the posterior margins of both acetabula and a line connecting the anterior and posterior margins of the acetabulum.

The adjustment to the APP and the measurement of the abovementioned parameters was performed by two different, independent raters and at two different time points. The measurements of the radiographs were compared with the results of the CT scan. For this purpose, the radiographic magnification as described above was considered before the comparison of the measurements. The measurement results of the radiographs were corrected by the magnification factor of the radiographs (110%) in order to make the results of both imaging procedures comparable.

### Statistical analysis

Statistical analysis was performed using Microsoft Excel 2010® (Redmond, WA, USA) and IBM SPSS Statistics® Version 22 (Armonk, NY, USA). Descriptive statistics were performed for the continuous measurement values (measurements in mm and ratios) including mean value, standard deviation and min−/max-values. Bland-Altman-Plots were used to compare the continuous measurement values and Cohen’s Kappa was applied for the categorical data. Intra- and inter-observer reliability was determined for the CT assessment by the intraclass correlation coefficient (ICC) and Cohen’s Kappa. An ICC of ≥0.8 indicates an adequate correlation. A Cohens Kappa of 0.41–0.60 shows a moderate, of 0.61–0.80 a good and of 0.81–1.00 an excellent correlation. For the comparison of the different positioning techniques with each other, a correlation analysis of the mean absolute differences was performed by a paired t test. All *p*-values were two-sided and *p*-values <0.05 were accepted to be statistically significant.

## Results

The descriptive statistics of the measurements with and without the use of the positioning devices (positioning I-III) showed a high variability (Table 1).

**Table 1:** Descriptive statistics: mean value ± standard deviation [min−/max-values]

	Positioning I	Positioning II	Positioning III	CT
Vertical distance symphysis-sacrococcygeal joint (in mm)	40.1 ± 18.5 [12.6–70.7]	35.3 ± 19.6 [11–73.8]	36.8 ± 22.2 [3.6–71.1]	25.4 ± 13.9 [8.3–46.1]
Horizontal distance symphysis-midpoint sacrococcygeal joint (in mm)	4.8 ± 3 [0–10.4]	4.1 ± 3.1 [0–10.4]	5.3 ± 4.1 [0–13.5]	3.8 ± 1.4 [1.7–6.1]
Vertical distance symphysis-tip of the coccyx (in mm)	13.5 ± 16.1 [− 16.2–33.3]	11.9 ± 15 [− 12.6–33.8]	8.2 ± 19.1 [− 23.4–34.2]	1 ± 16.3 [− 29.2–20.5]
Vertical distance symphysis-lower end of the sacroiliac joint (in mm)	83.9 ± 19.1 [63–123.3]	80.7 ± 15.9 [63.9–117]	79.2 ± 17.4 [59.4–98.6]	68.1 ± 15.7 [49.8–100.8]
Ratio vertical/horizontal diameter pelvic foramen	0.6 ± 0.1 [0.48–0.92]	0.6 ± 0.1 [0.45–0.75]	0.6 ± 0.1 [0.46–0.75]	0.5 ± 0.1 [0.4–0.72]
Ratio vertical/horizontal diameter obturator foramen right	1.0 ± 0.2 [0.54–1.41]	1.0 ± 0.2 [0.69–1.31]	1.0 ± 0.2 [0.74–1.49]	1.2 ± 0.1 [0.96–1.39]
Ratio vertical/horizontal diameter obturator foramen left	1.1 ± 0.3 [0.71–1.56]	1.1 ± 0.2 [0.84–1.36]	1.1 ± 0.2 [0.78–1.63]	1.2 ± 0.2 [0.77–1.54]

**Fig. 5:**
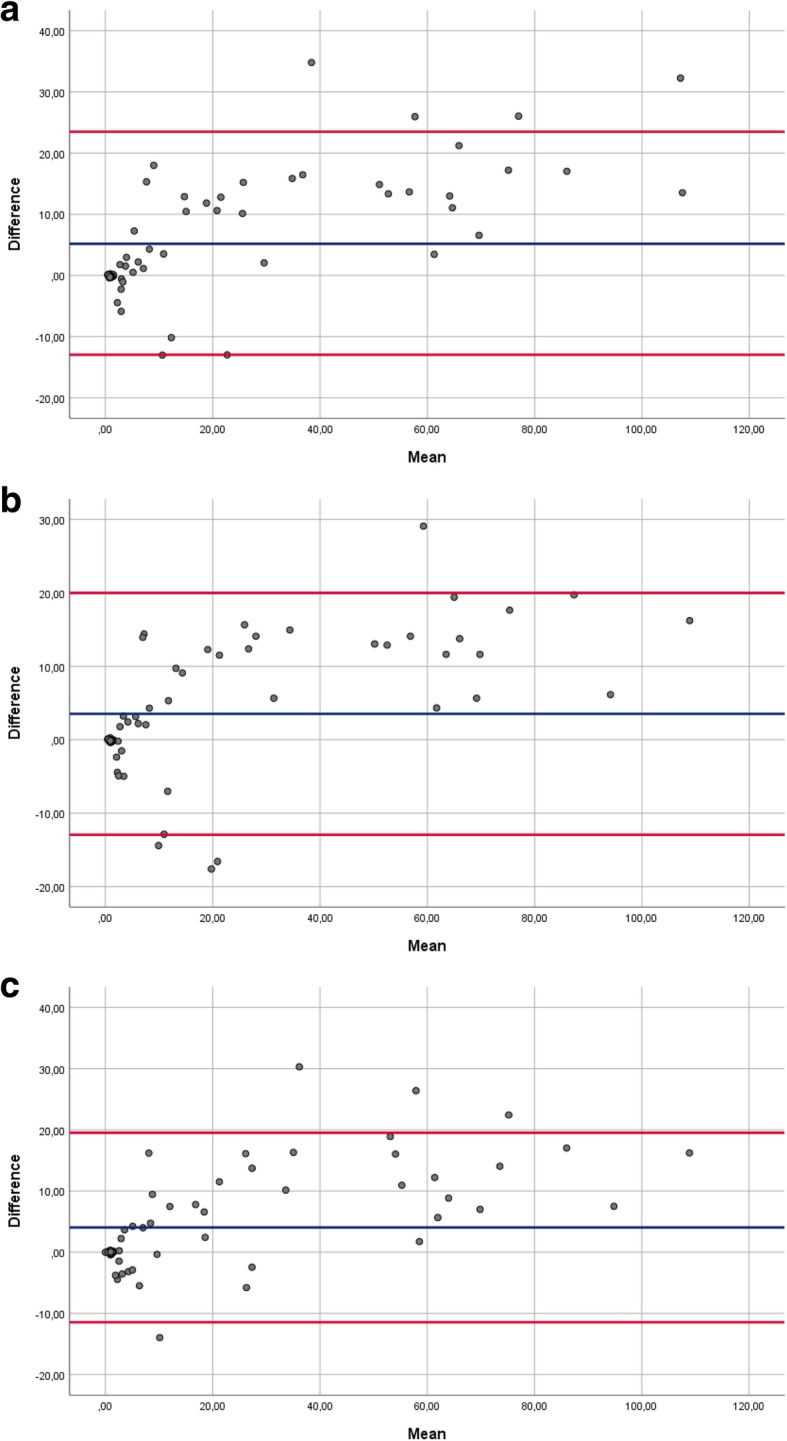
Bland-Altman-Plots showed a low correlation for the continuous measurement values between the radiographs of **a** positioning I, **b** positioning II, **c** positioning III and the corresponding CT scans (upper red line: + 1.96xSD, blue line: mean of the difference, lower red line: − 1.96xSD)

The Bland-Altman-Plots and Cohen’s Kappa did not show a high correlation for the continuous and categorical measurement values between the radiographs of positioning I-III and the corresponding CT scans (Fig. 5 and Table 2).

**Table 2:** Cohen’s Kappa values showed a low correlation for the categorial measurement values between the radiographs of (a) positioning I, (b) positioning II, (c) positioning III and the corresponding CT scans (COS: cross-over sign, ISS: ischial spine sign)

Cohen’s Kappa	(a)	(b)	(c)
COS left	0.54	0.50	0.50
COS right	0.24	0.33	0.45
ISS left	0.29	0.29	0.29
ISS right	0.42	0.30	0.42

The intra- and inter-observer reliability was good for the categorical measurements of the CT assessment (Cohen’s Kappa 0.78 and 0.69) and adequate for the continuous measurements (ICC 0.99 and 0.98). The use of positioning devices did not lead to more accurate AP pelvis radiographs compared to non-instrumented positioning. The results of both positioning devices were not superior to the results of the non-instrumented positioning technique (Table 3). Also, the measurements which were achieved by the standard method did not show a high correlation with the results of the CT scan.

**Table 3:** Correlation analysis of the mean absolute differences was performed by a paired t test for the comparison of the different positioning techniques among each other: (a) positioning I versus II, (b) positioning I versus III, (c) positioning II versus III

Measurement value	Correlation (a)	Significance (a)	Correlation (b)	Significance (b)	Correlation (c)	Significance (c)
(1)	0.69	0.02	0.77	0.01	0.55	0.08
(2)	0.32	0.33	0.07	0.84	0.41	0.21
(3)	0.35	0.39	0.12	0.76	0.17	0.66
(4)	0.04	0.91	0.31	0.38	0.39	0.26
(5)	0.02	0.96	0.21	0.56	0.42	0.22
(6)	0.54	0.08	0.13	0.71	0.38	0.24
(7)	0.62	0.06	0.62	0.06	0.77	0.01

## Discussion

The main results of this study are that both instruments used for positioning of the pelvis for standardized AP radiographs did not improve the accuracy of the radiographs compared to non-instrumented positioning. Also, the results of the non-instrumented pelvis positioning technique showed weak correlation with the results of the CT scan as the standard of reference. This confirmed our clinical suspicion that the current non-instrumented positioning method for AP pelvis radiographs had a limited accuracy and that there is an explicit need to improve the technique.

Therefore, it is still crucial to check the AP pelvis radiographs for a correct orientation of the pelvic bone with respect to tilt and rotation before a reliable diagnosis can be made. The most important aim is to avoid false-negative or false-positive diagnoses due to an incorrect pelvic positioning for the acquisition of the radiograph. Simple aids were the evaluation of the depiction of pelvic foramen and the obturator foramen and the assessment of the vertical and horizontal distance between the symphysis and the sacrococcygeal joint. Tannast et al. (Tannast et al. 2006) described that the distance of the upper border of the symphysis and the sacrococcygeal joint showed the strongest correlation with the pelvic tilt. Siebenrock et al. (Siebenrock et al. 2003) suggested a distance between the symphysis and the sacrococcygeal joint of 32 mm in men and 47 mm in women to achieve the neutral pelvic tilt. In case of incorrect tilted or rotated AP pelvis radiographs the examiner should repeat the radiographic examination with an improved pelvic orientation. Most recently, Schwarz et al. (Schwarz et al. 2018) described a reliable method for evaluating the pelvic tilt in AP radiographs. They analysed the height of the lesser pelvis and the obturator foramen in simulated AP radiographs for ten male and ten female pelvises in defined tilt positions. Corresponding tilt ratios were determined by the means of these measurements. The authors found that a tilt ratio of 0.5 defined a neutral pelvic tilt when the height of the lesser pelvis is twice the height of the obturator foramen.

Until the present day, no positioning device for AP pelvis radiographs has been established in the clinical practice and no comparable instruments were found in the recent literature. Nevertheless, there is a need for more precise and standardized radiographs of the pelvis. Wassilew et al. (Wassilew et al. 2012) analysed the diagnostic accuracy of the COS and PWS as signs for acetabular retroversion in 50 hips of patients who suffered from FAI. The results showed that these radiographic signs determined on AP pelvis radiographs were limited by pelvic tilt. Monazzam et al. (Monazzam et al. 2013) described that pelvic tilt and rotation influenced the assessment of acetabular overcoverage on AP pelvis radiographs and EOS® images. They concluded that a standardized pelvis radiograph is essential to prevent an inaccurate measurement of hip parameters due to excessive pelvic tilt or rotation. The results of the present study showed that the current non-instrumented position technique is lacking accuracy and underlined the need to improve its precision.

Siebenrock et al. (Siebenrock et al. 2003) and Tannast et al. (Tannast et al. 2005; Tannast et al. 2006) also analysed the influence of pelvic tilt and rotation on the assessment of the acetabular version on radiographs. In order to reduce the effect of pelvic tilt and rotation on radiographs, they developed a computer-based model and software that allows to edit the conventional radiographs (Tannast et al. 2008). Furthermore, they analysed which radiographic hip parameters were significantly influenced by pelvic tilt and rotation. Ghostine et al. (Ghostine et al. 2017) performed a 3D reconstruction based on biplanar radiographs to evaluate the influence of pelvic orientation on the reliability of pelvic parameters. The measurements performed with the 3D reconstruction were less sensitive to incorrect patient axial positioning compared to conventional radiographs. Nevertheless, patient axial malrotation should not be more than 10° for this analysis. In contrast to these post-editing techniques, the approach of this study was to achieve exact, reproducible and standardized AP pelvis radiographs that improve the reliable assessment of the spatial acetabular orientation and that do not have to be corrected for pelvic tilt and rotation with complex and cost-intensive software or 3D reconstruction. Furthermore, unnecessary repetitions of the radiographs or even misdiagnosis due to incorrect pelvic positioning should be reduced. For the construction of the instruments, this study used the APP concept which was introduced by Lewinnek et al. (Lewinnek et al. 1978). The APP is formed by the anterior superior iliac spines and the tubercle of the pubic symphysis. It should be orientated parallel to the radiographic film and vertically to the central beam to achieve a standardized orientation of the pelvis. Previous studies also used this concept to ensure a constant pelvic position for the assessment of the acetabulum (McKibbin 1970; Wassilew et al. 2012). The underlying methods of both devices respected these anatomical landmarks for the application. Both instruments are radiolucent, quick and easy to use, simple, robust, low costs causing and applicable in every radiology department.

However, in contrast to our hypothesis, both instruments that were used for positioning of the pelvic specimens for standardized AP radiographs did not improve the accuracy of the radiographs for reliable assessment of the acetabular orientation compared to the non-instrumented positioning. Therefore, the use of these positioning devices in the current state will not increase the quality of the AP pelvis radiographs in clinical practice.

Reasonable explanations could be the difficulty to define and to mark the relevant anatomic landmarks in an exact, reproducible and stable way. The underlying methods used for the instruments to ensure the correct pelvic orientation might be not precise enough. Therefore, a more accurate marking of the relevant landmarks with the use of laser, navigation or optical tracker might be mandatory. An incorrect position of the central beam might have also affected the results of the radiographic imaging. The results of the study of Schwarz et al. (Schwarz et al. 2018) indicated that the vertical central beam offset from the symphysis shows a high effect on the pelvic tilt in AP radiographs and should be taken into consideration.

Limitations of the study are that the results were achieved with cadaveric specimens and cannot be directly transferred to the clinical practice with living patients. In the present study, the positioning technique without any device (non-instrumented) is affected by the obviously visible bony APP landmarks. This is in contrast to the typical clinical setting where those relevant bony landmarks were not directly visible for the operator. The correct definition of the anatomic landmarks that were used for the placement of the positioning devices might be easier in cadaveric specimens than in real clinical settings. Especially in obese patients, the application of these devices and the reliable palpation of the APP landmarks could be difficult and challenging. Thus, the possibility of an extensive clinical use of the devices is limited. The patient’s movements during the examination might additionally influence the results. Other methods to objectify the rotation of the APP as recently described by Schwarz et al. (Schwarz et al. 2018) were not applied in this study. The additional assessment of tilt ratios would have been useful and would have improved the methodological strength of this study. Furthermore, the radiographs were performed in a static and supine position and did not represent a functional position of the pelvis. In contrast to the functional coronal plane, AP radiographs in the supine position did not respect the individual pelvic tilt. Therefore, important information concerning the pelvic tilt get lost on AP radiographs in this position. The number of pelvic specimens is acceptable, but limited to eleven samples. Further studies with the use of improved positioning techniques (laser, navigation or optical tracker system) and a larger number of specimens are needed to solve these problems.

## Conclusions

Simple mechanical positioning devices do not provide standardized AP pelvis radiographs for reliable assessment of the acetabular orientation. Both devices were not superior to the non-instrumented method of pelvic positioning for AP pelvis radiographs. Also, the measurements which were achieved by the non-instrumented method did not show a high correlation with the results of the CT scan.

## Data Availability

The datasets used and/or analysed during the current study are available from the corresponding author on reasonable request.

## Abbreviations

AP: anteroposterior
APP: anterior pelvic plane
COS: cross-over sign
CT: computed tomography
FAI: femoroacetabular impingement
ICC: intraclass correlation coefficient
ISS: ischial spine sign
MR: magnetic resonance
PWS: posterior wall sign
